# Potential role of cannabidiol in Parkinson’s disease by targeting the WNT/β-catenin pathway, oxidative stress and inflammation

**DOI:** 10.18632/aging.202951

**Published:** 2021-04-13

**Authors:** Alexandre Vallée, Jean-Noël Vallée, Yves Lecarpentier

**Affiliations:** 1 Department of Clinical Research and Innovation (DRCI), Foch Hospital, Suresnes 92150, France; 2 Centre Hospitalier Universitaire (CHU) Amiens Picardie, Université Picardie Jules Verne (UPJV), Amiens 80054, France; 3 Laboratoire de Mathématiques et Applications (LMA), UMR CNRS 7348, Université de Poitiers, Poitiers 86000, France; 4 Centre de Recherche Clinique, Grand Hôpital de l’Est Francilien (GHEF), Meaux 77100, France

**Keywords:** cannabidiol, WNT/β-catenin pathway, inflammation, oxidative stress, Parkinson's disease

## Abstract

Parkinson’s disease (PD) is a major neurodegenerative disease (ND), presenting a progressive degeneration of the nervous system characterized by a loss of dopamine in the substantia nigra pars compacta. Recent findings have shown that oxidative stress and inflammation play key roles in the development of PD. However, therapies remain uncertain and research for new treatment is of the utmost importance. This review focuses on the potential effects of using cannabidiol (CBD) as a potential therapeutic strategy for the treatment of PD and on some of the presumed mechanisms by which CBD provides its beneficial properties. CBD medication downregulates GSK-3β, the main inhibitor of the WNT/β-catenin pathway. Activation of the WNT/β-catenin could be associated with the control of oxidative stress and inflammation. Future prospective clinical trials should focus on CBD and its multiple interactions in the treatment of PD.

## INTRODUCTION

Parkinson’s disease (PD) is a main neurodegenerative diseases (ND), in that degeneration of the nervous system is marked by a loss of dopamine in the substantia nigra pars compacta (SNPC). This disease is localized in the brain and/or in the spinal cord, patients remain without any symptoms for a considerable duration [1, 2]. PD causes are still unknown but the presence of Lewy bodies (clumps of a-synuclein and ubiquitin proteins in neurons) was shown from the early steps of the condition. PD shows tremor symptoms, instability in postural, rigidity and postural instability. These symptoms appear only when majority of the dopaminergic (DAergic) cells has been lost in the SNPC, meaning that the smooth, which are the regulation control of striatal motor circuits, was also lost [3]. Non-motor symptoms, including depression and rapid eye movement (REM)-associated sleep behavior disorder (RBD), could involve the initiation of PD.

Aging is a main risk marker of neurodegeneration, as it may dysregulate the different signaling that modulate homeostasis processes in cells. Cells with neurodegeneration are the sites of numerous molecular and cellular dysregulation [4]. Numerous metabolic processes, including inflammation and oxidative stress (OS), could involve to neurodegenerative mechanisms. PD highlights a metabolic reprogramming involving stimulation of OS and inflammation [5, 6]. For a few years now, the WNT/β-catenin pathway was shown to be a major signaling systems implicated in PD [7, 8] and its dysregulation an early sign in the development of the condition [9].

Currently, drug therapies the main efficient and widely utilized treatments in PD are the use of levodopa, DA agonists, amantadine, monoamine oxidase B (MAO-B) inhibitors [10], catechol-O-methyltransferase (COMT) negative regulators [11], and many anticholinergic therapies. As physiotherapy, the nuclear destruction and stimulation of deep brain [12] are novel strategies, showing a great interest. Moreover, adjuvant therapies also are interesting for re- mission and preliminary therapy in PD. Although these drugs can counteracted many symptoms of PD to some extent, these cannot counteract PD development and can lead to many adverse effects. Currently, cannabidiol (CBD) is one of the main interesting therapy way for NDs [13, 14].

Cannabidiol (CBD) is a non-psychotomimetic phytocannabinoid derived from the *Cannabis sativa* plant. The plant possesses many therapeutic properties for a range of neurodegenerative diseases [13–15] and, in the few years, CBD has presented increasing interest as a possible anxiolytic therapy [16–18]*.* CBD decreases the stimulation of GSK3-β, an negative modulator of the WNT/β-catenin pathway [19], and has been found to suppress inflammatory signaling [20, 21] and oxidative stress [22]. The present review focuses on these metabolic mechanisms and the potential beneficial effects of cannabidiol (CBD) as part of a therapeutic strategy in PD.

## Parkinson’s disease and oxidative stress

Several findings have documented the stimulation of OS in PD [23]. Mitochondrial deregulation was shown in PD by increasing energy production and then, the release of reactive oxygen species (ROS) [24]. A decrease in mitochondrial activity involves cell damage and death through a decrease in energy production due to the enhancement of OS [5, 25]. OS and mitochondrial depletion have been found to be correlated with dementia and cell death [26–28]. A decrease in in the activity of the respiratory chain in the SNPC of a patient with PD is correlated with an augmentation in ROS production and apoptosis initiation [24, 29, 30].

Body can produce free radicals of oxygen for oxidative metabolism. In the aerobic respiration, molecular oxygen (O2) is diminished to water molecules in mitochondria. Through this phenomenon, O2, H2O2 and OH are generated by a leakage of oxygen [6]. Phagocytic cells, in response to inflammation and infection, produce high rates of NO, O2 and H202 to protect the human body and thereby diminish this infection. However, the radicals generated could damage cells [31].

Many enzymes, such as monoamine oxidase (MAO), L-amino acid oxidase and tyrosine hydroxylase, are implicated in metabolism of dopamine and in ROS production [32]. ROS production is also generated by inflammation. However, several types of signaling activity can act together with ROS production. The ROS-induced proteins aggregation could lead to inflammatory process in microglia [33]. Four processes enhanced in PD are associated with inflammation and OS: stimulation in iron rates, the diminution in glutathione (GSH) rates, the decrease of 26S proteasomal function and the deregulation of mitochondrial complex I regulation [34, 35]. During the physiologic stage, MAO generates H_2_O_2_, but during PD development, H_2_O_2_ is changed into hydroxyl radicals (OH) through iron by the Fenton reaction. Then, H_2_O_2_ and OH enhance OS [36]. In the PD cytosol, H_2_O_2_ and OH oxidized GSH [37], involving leakage of GSH. The GSH leakage generates the transformation of glutamate and cysteine into peptides called glutamyl and cysteinyl. These peptides have a adverse effect on dopaminergic cells by linking the membrane of cells and by increasing ROS production in dopaminergic neurons. They also diminish the stimulation of the mitochondria complex I, which leads to OS and ROS production [38]. DAergic cells are not available to bind misfolded proteins because of the impair in proteasomal mechanisms [39]. OS involves the carbonylation of proteins, leading to an unrepairable and irreversible change. Carbonylation is a phenotype of senescence of cells enhancing the aggregation of proteins. In PD, proteins aggregation is a main pathological feature of nigrostriatal DAergic neurons. Proteins aggregation leads to neuroinflammation and OS [40].

## Parkinson’s disease and inflammation

Recent PD studies have presented that inflammation has a main action [41] by activating the apoptosis pathways in dopaminergic cells [42, 43]. The association between inflammation and PD is 2-way; inflammatory processes enhance dopaminergic cells death, death of DAergic cells, in a vicious loop, can also stimulate inflammation [44]. Furthermore, inflammatory markers lead to OS, involving DAergic cells to stimulate death signaling [45]. In PD, many inflammatory markers, such as microglia, show a major action [46]. Microglia stimulation can activate their pro-inflammatory enzymes (including inducible nitric oxide synthase and COX) and releasing of inflammatory cytokines (including tumor necrosis factor-α (TNF-α), interferon-γ (IFN-γ), C-X-C motif chemokine ligand 12 (CXCL12), interleukin (IL)-6 and IL-1β) [47]. In microglia, the NF-κB pathway plays a main action in the generation of these inflammatory cytokines [48]. TNF-α stimulates apoptosis through the TNF-R1 receptor death domain which activates the caspases 1 and 3 [49]. TNF-α decreases c-Rel–NF-κB. c-Rel–NF-κB plays a neuroprotective function by inhibiting apoptosis via the B-cell lymphoma-extra-large signaling in DAergic neurons [48]. PD shows increased rates of CXCR4 (named fusin) expression and its ligand CXCL12. The dimer composed by CXCR4-CXCL12 stimulates caspase 3, enhancing apoptosis and then neural cell death [50, 51]. The dimer of IFN-γ–IFNGR pathway leads to the phosphorylation of the leucine-rich repeat kinase 2 (LRRK2) protein [52]. In microglia and in DAergic neurons, LRRK2 binds to several cellular mechanisms. Stimulated LRRK2 downregulates expression of c-Rel–NF-κB. Then, inflammatory process is stimulated by decreasing c-Rel–NF-κB activity [53, 54]. LRRK2 stimulation can lead to the initiation of tau oligomers, stimulating cell death signaling [55, 56]. In cells, LRRK2 modulates several vesicle trafficking and its up-regulation is correlated with an increase in inflammatory cytokines [57].

## WNT/β-catenin pathway

The WNT name is comes from “*Wingless drosophila melanogaster*” and its mouse homolog *“Int”.* The WNT/β-catenin pathway is implicated in many signals and molecular processes, including cell proliferation, embryogenesis, cell migration and cell polarity, apoptosis, and organogenesis [58]. Nevertheless, the WNT/β-catenin pathway can be deregulated during numerous pathological states, such as inflammation, neurological disorders, metabolic diseases, tissue fibrosis and cancer processes [59].

The WNT pathway belongs to the family of secreted lipid-modified glycoproteins [60]. WNT ligands are secreted by both immune cells and neurons located in the CNS [61]. Modulation of the WNT/β-catenin pathway implicates metabolic pathways, embryony development, cell fate, and epithelial-mesenchymal transition (EMT). WNT/β-catenin pathway deregulation leads to numerous NDs, such as PD [6, 62–64]. At the transcriptional level, WNT signaling is primarily mediated by a family of transcription factors known as the β-catenin/T-cell factor/lymphoid enhancer factor (TCF/LEF). The cytoplasmic accumulation of β-catenin is generated by the complex “*AXIN, tumor suppressor adenomatous polyposis coli (APC), and glycogen synthase kinase-3 (GSK-3β)*”. In WNT ligands absence, this complex enhances to phosphorylate cytoplasmic β-catenin and involves its proteasomal degradation. In presence of the WNT ligands, β-catenin binds to Frizzled (FZL) and LDL receptor-related protein 5/6 (LRP 5/6), thereby stopping the complex and preventing β-catenin proteasomal degradation. β-catenin translocates to the nucleus to bind with TCF/LEF. This in turn activates WNT target genes [65–67].

**Figure 1:**
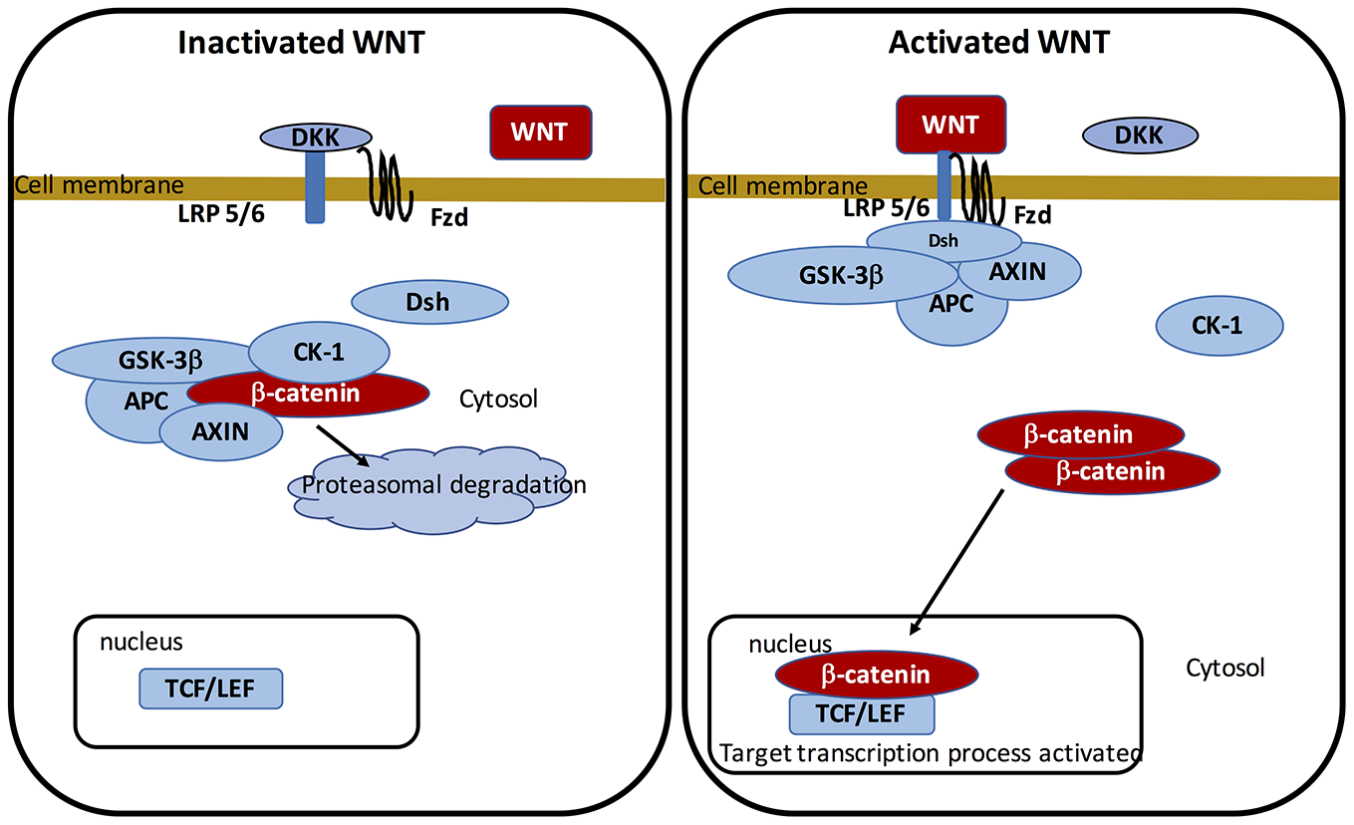
**On-state and off-state activation of the WNT pathway.**

GSK-3β is a major negative modulator of the WNT/β-catenin pathway [68–73]. As an intracellular serine-threonine kinase, GSK-3β is a controller of the WNT/β-catenin pathway [74]. GSK-3β is implicated in the modulation of numerous pathophysiological pathways, including cell membrane pathway, cell polarity, and inflammatory process [75–77]. GSK-3β acts by downregulating cytoplasmic β-catenin and stabilizes it to activate its nuclear translocation. Inflammatory process is an age-related mechanism correlated with the activation of GSK-3β activity and the decrease of the WNT/β-catenin pathway [78] (Figure 1).

## Parkinson’s disease and WNT/β-catenin pathway

Dysregulation in the WNT/β-catenin pathway is intricately involved in the development of PD [7, 8]. WNT/β-catenin pathway deregulation is an initiating event in PD [9]. Numerous cell biological roles damaged in PD are modulated by the WNT/β-catenin pathway, including axonal function, microtubule stability and membrane trafficking [79, 80]. The mesencephalic dopaminergic neuron-astrocyte interplay is modulated by WNT-1 controlled Frizzled-1/β-catenin pathway [81]. In normal conditions, LRRK2 binds to the WNT/β-catenin pathway and Dishevelled (DSH) proteins to downregulate the β-catenin destruction complex and to increase the WNT/β-catenin pathway [9]. In majority, PD is an idiopathic disease. Nevertheless, in familial Parkinsonism, genes are generally associated with PARK genes. PARKs mutations, codifying for LRRK2, have been observed to be an etiology of PD family forms [82]. LRRK2 mutations decrease the LRRK2-LRP5/6 binding affinity and are associated with a decrease in WNT pathway activity [83]. PARK2 gene encodes the E3 ubiquitin ligase Parkin. Parkin genetic damages are involved in PD initiation and operate as β-catenin inhibitors leading to β-catenin ubiquitination and proteasomal degradation [8]. In PD, DKK1 and GSK-3β are increased [84]. PD mice models present an interplay between inflammation, OS and the WNT/β-catenin signaling [85].

## Oxidative stress and WNT/β-catenin pathway

FoxO (Forkhead box class O) transcription factors are important intracellular modulators of numerous molecular pathways, such as glucose production and cell response to OS [86]. ROS production is correlated with the diminution of the WNT/β-catenin pathway by diverting β-catenin from TCF/LEF to FoxO [87]. This leads to the accumulation and binding of β-catenin to FoxO as a co-regulator, and in stimulating FoxO nuclear transcriptional activity [88, 89]. FoxO stimulates apoptotic genes expression [90–92]. FoxO3a interrupts the cell-cycle by activating the production of the cyclin-dependent kinase inhibitor p27 kip1 and the diminution of expression of cyclin D1 [93, 94]. The stimulation of FoxO induces of apoptosis [95]. Nevertheless, the increase of the WNT/β-catenin pathway can decrease FoxO3a in the cytoplasm to counteract mitochondrial membrane permeability loss, release of cytochrome c, phosphorylation of Bad, and the stimulation of caspases. Stimulation of the WNT/β-catenin pathway also activates OS and ROS production [96].

## Inflammation and WNT/β-catenin pathway

The activation of the WNT/β-catenin pathway decreases inflammation and enhances neuroprotection through several interplays between microglia/macrophages and astrocytes [81, 97].

Numerous findings have observed an interplay between the WNT/β-catenin and NF-κB pathways, major markers of inflammatory process [98]. The NF-κB transcription factor family comprises 5 compounds in the cytoplasm under unactuated steps: NF-κB 1 (p50/p105), NF-κB 2 (p52/p100), RelA (p65), RelB and c-Rel [99]. β-catenin complexes with RelA and p50 to decrease NF-κB signaling activity [100]. Furthermore, by binding to the PI3K, β-catenin decreases the activity of NF-κB pathway [101]. The downregulatory role of β-catenin on NF-κB pathway has been shown in several cellular signaling processes, including fibroblasts, epithelial cells, hepatocytes and osteoblasts [98]. Moreover, GSK-3β stimulation activity inhibits the β-catenin and then, a stimulation of NF-κB pathway [102]. The possible protective role of β-catenin is caused by the stimulation of the PI3K/Akt pathway and the decrease in the TLR4-driven inflammatory response [103]. NF-κB pathway stimulation inhibits the β-catenin/TCF/LEF complex by increasing LZTS2 [104]. DKK1, a negative modulator of the WNT pathway, is a target gene of the NF-κB pathway involving a negative interplay decreasing the β-catenin pathway [105]. Stimulated β-catenin downregulates the NF-κB-mediated transcription of pro-inflammatory genes. This phenomenon is directly modulated by the activity of GSK-3β [106, 107].

## Cannabidiol

Cannabinoids provide from a heterogeneous group of components characterized by 3 major components: endogenous, synthetic and phytocannabinoids [108, 109]. CBD is a non-psychotomimetic phytocannabinoid derived from the *Cannabis sativa* plant. The *Cannabis sativa* plant generates more than sixty-six components such as delta9-tetrahydrocannabinol (THC), causing psychological effects, and CBD, the main non-psychotomimetic component in the *Cannabis sativa* plant [110]. CBD presents no interaction with blood pressure or body temperature and no association with psychomotor psychological functions such as THC [111]. CBD attenuates damages in brain correlated with neurodegenerative processes. Human bodies could tolerate high doses of CBD [111]. Furthermore, CBD can interact with synaptic plasticity and induce neurogenesis mechanism. The mechanisms of CBD effects remain unclear but seem to have several pharmacological targets. Traditional medicines used *Cannabis sativa* for centuries. CBD, a major components of *Cannabis sativa*, has recently presented considerable interest for its potential role with respect to many neuropsychiatric disorders [112]. CBD presents a large spectrum of possible therapeutic properties, including anxiolytic, antidepressant, neuroprotective, anti-inflammatory and immunomodulatory processes [109]. Cannabinoids are a novel class of drugs due to their possible role in treating neuropsychiatric diseases [13], including schizophrenia, epilepsy, addiction and neonatal hypoxic-ischemic encephalopathy [113]. In schizophrenia, CBD stimulates the WNT/β-catenin and PI3K/Akt pathways to lead to therapy actions [14, 114, 115].

OS contributes to neurodegeneration. Thus, neuron cells present a functional or sensory loss in neurodegeneration. For life, even if oxygen is needed, an unbalanced metabolic process and an increased production of reactive oxygen species ends up in several NDs, including AD and PD. Free radicals lead to damages in protein and DNA, stimulate inflammation processes and apoptosis [116]. Some findings have shown that secondary plant metabolites, from medicinal herbs, could show lead components for medication production for inflammation and OS therapy, leading to protect from loss in neuronal cell [117]. Among them, CBD could be a prototype for anti-inflammatory and antioxidative therapy for these diseases where inflammation and OS have major actions in their etiologies and initiation [118].

In the CNS, CBD has been shown to possess anti-inflammatory actions, thus being useful for neuro-inflammatory diseases [119], and therapy of spasticity and pain [120]. Based on its anticonvulsant roles, CBD has been used as a therapy for epilepsy [121], and also for the therapy for sleep disorders [122] due to its capability to control serotonin transmission [123]. CBD possesses interesting roles for psychiatric disorders, such as schizophrenia [124], but it also presents other possible actions, such as anxiolytic and antidepressant roles [125, 126]. The neuroprotective action of CBD for the management of certain other NDs has also been investigated in different studies that have yielded many positive results [13].

## Parkinson’s disease and cannabidiol

Recent clinical investigations have presented the interest of using CBD for its antiparkinsonian properties [127–131]. CBD can significantly reduce 6-OHDA-induced neurotoxic actions in mice, and this neuro-protective role could be controlled through cannabinoid receptor-independent anti-inflammatory and antioxidant actions [127]. CBD can also target and reduce the different inflammatory factors, including COX-2 and NF-κB. These factors have been found to be blocked by the CBD effect on PPARγ receptors [131, 132]. Moreover, CBD can reduce DA depletion and slow down the increase in OS [13, 133]. The latter evidence suggests that CBD has antioxidant properties and can diminish the nigrostriatal dopaminergic neurodegeneration fibers observed in PD [134]. Furthermore, CBD presents a high possible antioxidant actions, compared to ascorbate, for cortical neurons treated with toxic glutamate concentrations [15]. The neuroprotective action was shown regardless of whether the insult was due to the stimulation of N-methyl-D-aspartate (NMDA) receptor, a-amino-3-hydroxy-5-methyl-4-isoxazolepropionic acid (AMPA) receptor, or kainate receptors and, it is not controlled by CB receptors since the CB antagonist is not damaged [135]. The recent result may present that CBD could be a possible antioxidant without psychotropic adverse effects, directly controlled by CB receptors.

## Stimulation of the WNT/β-catenin pathway by cannabidiol: a possible therapeutic strategy (Figure 2)

**Figure 2:**
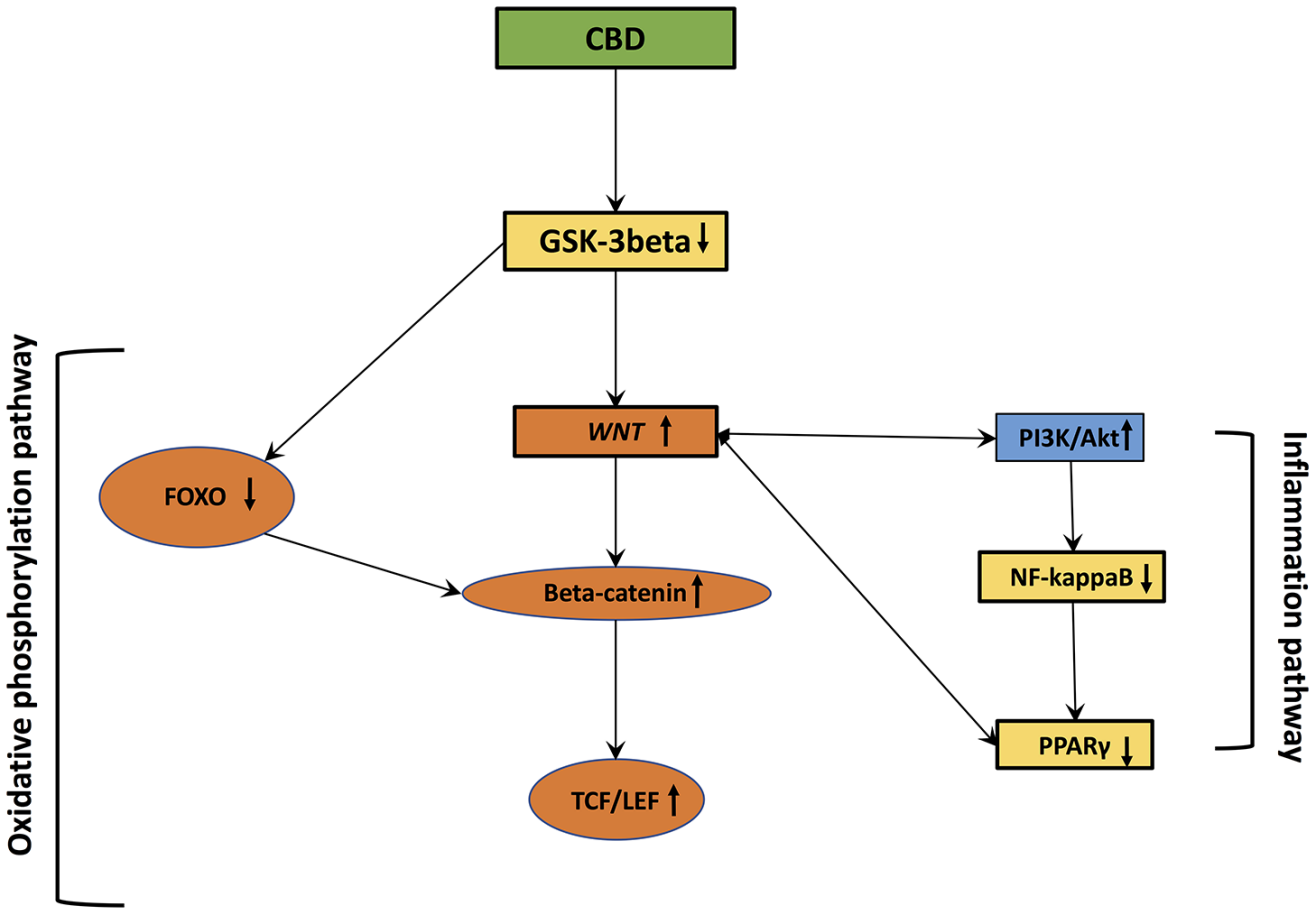
**Cannabidiol interactions with oxidative stress and inflammation.**

A study have observed that mutant OCD mouse models presented stimulated GSK-3β activity, suggesting that GSK-3β downregulation can provide a therapy for perseverative behavior [136].

Dysfunction of GSK-3β is implicated in the pathogenesis of several disorders, such as neuropsychiatric disorders [137]. GSK3β is known to be the main inhibitor of the WNT/β-catenin pathway [72, 138–140]. GSK-3β downregulates the WNT/β-catenin pathway by inhibiting β-catenin cytoplasmic stabilization and its nuclear translocation [83]. Moreover, many findings have observed a link between neuro-inflammation and the augmentation of the GSK-3β and a decrease in the activity of the WNT/β-catenin pathway and the Akt pathway [68].

CBD downregulates expression of GSK-3β through the promotion of PI3K/Akt signaling [141, 142]. PI3K/Akt signaling regulates GSK-3β activity [143]. Cannabinoids control the PI3K/Akt/GSK-3β axis [144, 145]. Gene coding for the PI3K/Akt pathway is increased in CBD-GMSCs (mesenchymal stem cells derived from gingiva treated by CBD) [141]. Diminution of β-catenin activity reduces the expression of PI3K/Akt pathway [146]. In schizophrenia, CBD stimulates the WNT/β-catenin and PI3K/Akt pathways to enhance therapeutic actions [13]. Cannabinoids can directly control the PI3K/Akt/GSK-3β axis [144, 145]. In GMSCs treated by CBD, genes coding for the PI3K/Akt signaling are increased [141]. CBD downregulates GSK-3β activity by stimulating PI3K/Akt pathway [141]. In neurons and glial cells, CBD can stimulate the PI3K/Akt pathway by interacting with CB1 receptor and, in a less manner in the immune system with CB2 receptor [147, 148].

CBD can reduce the activity of the pro-inflammatory factors COX-2 and NF-κB. These effects are stopped by the combination of CBD and PPARγ receptors. The neuroprotective effects of CBD are generated by anti-inflammatory actions modulated by both CB1 and PPARγ [149]. By interacting with PPARγ, CBD can activate the canonical WNT pathway to reduce inflammation and OS [14].

## Oxidative stress and cannabidiol

The energy production and metabolism of glucose implicated in OS are regulated by the FOXO transcription factors [86]. This relationship between β-catenin and FOXO leads to the promotion of cell quiescence and cell cycle stop. β-catenin interrupts its complex with TCF/LEF by binding with FOXO [87]. β-catenin did not lead to nuclear translocation to and accumulates in the cytoplasm to inactivate the WNT/β-catenin pathway [88, 89].

CBD can reduce the redox balance through the modification of both the rate and activity of oxidants and antioxidants [22]. CBD stops free radical chain reactions through the capture of free radicals and then by reducing their activities [150]. CBD downregulates the oxidative conditions through the prevention of the initiation of superoxide radicals, produced by xanthine oxidase (XO) and NADPH oxidase (NOX1 and NOX4) [151, 152]. Moreover, CBD can enhance the diminution in NO levels [153]. CBD also diminishes ROS production through the chelation of the transition metal ions implicated in the Fenton reaction to enhance hydroxyl radicals [154]. CBD acts on the antioxidant BHT (butylated hydroxytoluene) to prevent dihydrorodamine oxidation in the Fenton reaction [15].

The antioxidant activity of CBD is characterized by the stimulation of redox-sensitive transcription factor associated with the Nrf2 (Nuclear factor-erythroid 2 related factor 2) [155], which controls the transcription of cytoprotective genes [156]. Superoxide dismutase (SOD) and the enzymatic activities of Cu, Zn and Mn-SOD, controlling superoxide radicals metabolic processes, are increased by CBD [157]. Glutathione peroxidase and reductase are also increased by CBD, decreasing the malonaldehyde (MDA) levels [158]. Enzymatic activities are altered during oxidative modifications of proteins. CBD, by targeting glutathione and cytochrome P450, can inhibit their biological activity and thus decrease oxidative stress [153, 159]. Moreover, through the decrease in ROS levels, CBD can prevent and protect non-enzymatic antioxidants [157], including vitamins A, E and C [160].

## Inflammation and cannabidiol

Cannabinoids present anti-inflammatory action by endogenous receptors, including CB1 and CB2 [161]. N-Oleoyl glycine (OLGly), a lipoamino acid, activates adipogenic genes including PPARγ, a marker of inflammation, and the expression of mRNA of the CB1 receptor. Inhibition of the CB1 receptor by SR141716 downregulates the actions of OLGly on PPARγ. Moreover, OLGly activates the Akt pathway to inhibit FoxO activity [162]. CBD can bind PPARγ [14, 163]. PPARγ is a major factor of inflammation through its interaction with NFκB. This binding acts on the ligand-binding domain of PPARγ and the Rel homology domain region of the p65 subunit of NFκB. Proteasomal degradation of p65 is caused by the Lys48-linked polyubiquitin of the ligand-binding domain of PPARγ [164]. Thus, PPARγ can modulate inflammation through the ubiquitination proteasomal degradation of p65 leading to the control of cyclooxygenase (COX2), TNF-α, IL-1β and IL-6 [14]. PPARs are ligand-activated transcription factors binding PPREs (PPAR-response elements), and are implicated in several dysregulated mechanisms, including cell differentiation, protein metabolisms, lipid metabolisms, carcinogenesis [165, 166], adipocyte differentiation, insulin sensitivity and inflammation [167, 168]. PPARγ ligands, including thiazolidinediones (TZDs), can diminish inflammation [169]. A negative crosstalk between PPARγ and the WNT/β-catenin pathway has been well documented [138, 170–172]. The PI3K/Akt pathway, enhancing by β-catenin [140, 171, 173–175], interacts through the phosphorylation of GSK-3β to decrease PPARγ [176]. PPARγ agonists inhibit β-catenin through the stimulation of GSK-3β activity [177]. PPARγ agonists activate DKK1 expression to decrease the WNT/β-catenin pathway [178]. Moreover, PPARγ agonists stimulate GSK-3β activity to inhibit β-catenin [177]. In parallel, β-catenin directly inhibits NFκB activity [106, 107].

## CONCLUSIONS

To date, few studies have studied CBD as a potential alternative therapeutic solution in treating PD. However, CBD is attracting increasing interest in this context because of its possible inhibitory effect on OS and inflammation and the fact that, at low doses there are few side effects. WNT/β-catenin pathway activity is diminished in the development of PD. By stimulating the WNT/β-catenin pathway, through the decrease of GSK-3β, CBD may be an integral part of an innovative therapeutic treatment of the condition. Future investigated studies should therefore focus on CBD and its many relationships with the development and treatment of PD.
